# Genetic factors increase the identification efficiency of predictive models for dyslipidaemia: a prospective cohort study

**DOI:** 10.1186/s12944-021-01439-3

**Published:** 2021-02-12

**Authors:** Miaomiao Niu, Liying Zhang, Yikang Wang, Runqi Tu, Xiaotian Liu, Jian Hou, Wenqian Huo, Zhenxing Mao, Zhenfei Wang, Chongjian Wang

**Affiliations:** 1 Department of Epidemiology and Biostatistics, College of Public Health, Zhengzhou University, 100 Kexue Avenue, Zhengzhou, 450001 Henan People’s Republic of China; 2 School of Information Engineering, Zhengzhou University, Zhengzhou, Henan People’s Republic of China

**Keywords:** Dyslipidaemia, Genetic risk score, Machine learning, Risk model, Predictive performance, Lipid level, Classifier

## Abstract

**Background:**

Few studies have developed risk models for dyslipidaemia, especially for rural populations. Furthermore, the performance of genetic factors in predicting dyslipidaemia has not been explored. The purpose of this study is to develop and evaluate prediction models with and without genetic factors for dyslipidaemia in rural populations.

**Methods:**

A total of 3596 individuals from the Henan Rural Cohort Study were included in this study. According to the ratio of 7:3, all individuals were divided into a training set and a testing set. The conventional models and conventional+GRS (genetic risk score) models were developed with Cox regression, artificial neural network (ANN), random forest (RF), and gradient boosting machine (GBM) classifiers in the training set. The area under the receiver operating characteristic curve (AUC), net reclassification index (NRI), and integrated discrimination index (IDI) were used to assess the discrimination ability of the models, and the calibration curve was used to show calibration ability in the testing set.

**Results:**

Compared to the lowest quartile of GRS, the hazard ratio (*HR*) (95% confidence interval (*CI*)) of individuals in the highest quartile of GRS was 1.23(1.07, 1.41) in the total population. Age, family history of diabetes, physical activity, body mass index (BMI), triglycerides (TGs), high-density lipoprotein cholesterol (HDL-C), and low-density lipoprotein cholesterol (LDL-C) were used to develop the conventional models, and the AUCs of the Cox, ANN, RF, and GBM classifiers were 0.702(0.673, 0.729), 0.736(0.708, 0.762), 0.787 (0.762, 0.811), and 0.816(0.792, 0.839), respectively. After adding GRS, the AUCs increased by 0.005, 0.018, 0.023, and 0.015 with the Cox, ANN, RF, and GBM classifiers, respectively. The corresponding NRI and IDI were 25.6, 7.8, 14.1, and 18.1% and 2.3, 1.0, 2.5, and 1.8%, respectively.

**Conclusion:**

Genetic factors could improve the predictive ability of the dyslipidaemia risk model, suggesting that genetic information could be provided as a potential predictor to screen for clinical dyslipidaemia.

**Trial registration:**

The Henan Rural Cohort Study has been registered at the Chinese Clinical Trial Register. (Trial registration: ChiCTR-OOC-15006699. Registered 6 July 2015 - Retrospectively registered).

**Supplementary Information:**

The online version contains supplementary material available at 10.1186/s12944-021-01439-3.

## Background

Dyslipidaemia is an important risk factor for cardiovascular disease (CVD) development [1]. Studies have shown that approximately 20% of atherosclerosis patients have either high triglyceride (TG) or low high-density lipoprotein cholesterol (HDL-C) lipid levels [2], while the incidence of heart disease and ischaemic stroke decreases accordingly with lower low-density lipoprotein cholesterol (LDL-C) levels [3]. Furthermore, numerous studies have demonstrated that elevated serum total cholesterol (TC), TG and LDL-C levels are closely related to the development of CVD and therefore could be used as an independent predictor of CVD [1, 4, 5]. In the past decade, the prevalence of dyslipidaemia has declined in developed countries [6], but that in China, the largest developing country, remains at a high level and continues to increase [7]. Reports have shown that a total of 9.2 million cardiovascular events will occur due to serum cholesterol levels in the Chinese population between 2010 and 2030 [8]. In addition, the age-standardized prevalence of adult dyslipidaemia was 32.21% in rural areas, with relatively low rates of awareness, treatment, and control (15.07, 7.23, and 3.25%, respectively) [9]. The above data indicated that the prevention of dyslipidaemia remains a huge public health problem in China, especially in rural areas. To date, the establishment of disease risk prediction models has received extensive attention globally in preventing diseases. Previously, validated disease prediction models for CVD and diabetes were developed based on the Framingham study [10, 11]. Moreover, some researchers have also focused on effective risk models for other diseases to help diagnosis and prevention [12–15]. However, few studies involved the prediction model for dyslipidaemia [16–19], and most of them were limited to specific populations such as children and adolescents to a certain extent.

Currently, there are different types of model building methods. Among them, the traditional statistical method suitable for survival data is Cox regression analysis [20]. Compared with traditional data processing methods, machine learning approaches in processing fuzzy data, random data, and nonlinear data have obvious advantages, especially for large-scale, complex, ambiguous information [21–23]. Given the known character of machine learning methods, a growing use of this burgeoning tool was reported especially with prediction issues.

As reported, the genetic risk score, which consists of multiple single nucleotide polymorphisms (SNPs), confers a strong prediction of cardiovascular risk, but each SNP alone does not contribute much [24]. Although the role of SNPs in dyslipidaemia were well known [25–27], no study has interpreted how polygenetic genetic risk scores (GRSs) affect dyslipidaemia when it is necessary to predict the risk of dyslipidaemia, especially in resource-limited areas. To that end, this study was constructed to set up a dyslipidaemia prediction model using different classifiers and to reveal the prediction performance of the model incorporating genetic factors in predicting the occurrence of dyslipidaemia in Chinese rural adults.

## Methods

### Study population

The study recruited participants from a cohort study in rural areas of Henan, called the Henan Rural Cohort Study, which has been registered in the Chinese clinical trials registry. The baseline examination and follow-up information have been previously described in detail [28]. In brief, the baseline investigation included a questionnaire interview, anthropometry measurements, and blood tests. The subjects were then asked about the occurrence of chronic diseases, including the type and duration of the disease, as well as the status of treatment and medication at the follow-up survey.

In this study, a total of 8268 subjects committed to genetic factors, and all subjects were tested for SNPs. In summary, 3596 individuals were finally analysed after excluding participants who 1) had dyslipidaemia at baseline; 2) were using lipid-lowering drugs; and 3) were missing important information about the key variables. Details can be found in the study population section of the flowchart of Fig. S1, which can be found in Supplementary Information.

### Data set

The 3596 study subjects were randomly divided into two sets of data, called the training set and the testing set, respectively. At a ratio of 7:3, 2517 study subjects were randomly selected for the training set, while 1079 study subjects were randomly selected for the testing set. Model building was performed in the training set, and the performance of the model was evaluated in the testing set.

### Definition of dyslipidaemia

As reported by the Guidelines for Dyslipidaemia in China [7], dyslipidaemia is defined as having greater than or equal to 1 of the following conditions: TC ≥ 6.2 mmol/L (240 mg/dL); TG ≥ 2.3 mmol/L (200 mg/dL); HDL-C ≤ 1.0 mmol/L (40 mg/dL); LDL-C ≥ 4.1 mmol/L (160 mg/dL); use of lipid-lowering drugs in the last two weeks. Notably, in this study, dyslipidaemia was determined using measured lipid levels. Because participants did not stop taking lipid-lowering drugs, those who used lipid-lowering drugs in the last two weeks were also considered dyslipidaemia patients, which was also used in a previously published article [9].

### Calculation of weighted genetic risk score (GRS)

Among tens of SNPs related to dyslipidaemia, 21 SNPs (rs10889353, rs11207995, rs7518497, rs780092, rs10045497, rs11216126, rs1160985, rs17119975, rs183786, rs328, rs3764261, rs3943077, rs4417316, rs507666, rs603446, rs651007, rs651821, rs6589566, rs662799, rs7396835, rs964184) were selected to calculate the weighted GRS (shown in Table S1). SNP genotyping was performed using a custom SNPscan™ kit (Genesky Biotechnologies Inc., Shanghai, China) [29]. Given the target population in this study, SNPs were selected based on the previously reported large GWAS for lipid profiles in East Asia, and then replicated in this cohort study.

The genotype of each SNP was assigned 0, 1, and 2 in ascending order of the number of alleles, and then Cox regression analysis was performed to obtain the effect value for each SNP (i.e., the *β* value in Table S1). The weighted GRS was the sum of the effect size of each SNP multiplied by the number of risk alleles. The mean value and standard deviation of the GRS were 1.329 and 0.337, respectively, ranging from 0.195 to 2.451.

### Statistical analysis

Statistical significance was inferred at a two-tailed value of *P* < 0.05. Differences in the characteristics of the dyslipidaemia and non-dyslipidaemia populations were compared using t-tests and chi-square tests. All subjects were divided into quartiles according to GRS. Taking Q1 as the reference group, the hazard ratios (*HR*s) of the remaining three GRS groups of subjects were calculated in the total population, as well as in the training and testing sets.

Cox regression was used to filter the predictors of the model. In the training set, all variables that have been reported as predictors were analysed using simple Cox regression (shown in Table S2). Then, those variables presenting a significant impact on dyslipidaemia entered the conventional models. The GRS mentioned above was then incorporated into the conventional models to constitute the conventional+GRS models. Cox regression also served as a traditional statistical classifier, which was performed as follows: in the training set, a multiple Cox analysis was performed to obtain effect values (*β*) for each predictor (i.e., the *β* values in Table 3), and then these *β* values were used to construct a Cox regression predictive probability model for the onset of dyslipidaemia in combination with the general formula of the Cox regression model. In the testing set, the Cox prediction model equation established in the training set was used to predict the risk of dyslipidaemia for each individual. In addition, artificial neural network (ANN), random forest (RF), and gradient boosting machine (GBM) were also employed to construct models, and the prediction model was trained and tested by 10-fold cross-validation with 100 repetitions during the iterative process.

The discrimination of models was assessed using the area under the receiver operating characteristic curve (AUC). The net reclassification index (NRI) and integrated discrimination index (IDI) were used to evaluate the improvement of predictive ability of the conventional models when adding GRS. The calibration of the models was assessed by calibration curves (See the model constitution and evaluation section in Fig. S1). Statistical analyses were performed with R 3.6.2 and Python 3.8.

## Results

### Baseline characteristics

The baseline characteristics of the dyslipidaemia and non-dyslipidaemia populations are shown in Table 1. The average age of all subjects was 50.49 ± 12.16 years. The incidence of dyslipidaemia was 44.38%. The differences in family history of diabetes, BMI, and lipid levels were statistically significant between dyslipidaemia and non-dyslipidaemia populations (all *P* < 0.05).

**Table 1:** Baseline characteristics of subjects with dyslipidaemia and without dyslipidaemia

Characteristic	Total (*n* = 3596)	Dyslipidaemia (*n* = 1596)	Non-Dyslipidaemia (*n* = 2000)	*P*-value
Age	50.49 ± 12.16	50.64 ± 12.09	50.38 ± 12.22	0.528
Family history of diabetes, n (%)	186(5.17)	100(6.27)	86(4.30)	0.008
Physical activity, n (%)				0.747
Low	1656(46.05)	724(45.36)	932(46.60)	
Moderate	810(22.53)	362(22.68)	448(22.40)	
High	1130(31.42)	510(31.95)	620(31.00)	
Body mass index (BMI), kg/m^2^	23.91 ± 3.36	24.58 ± 3.38	23.38 ± 3.24	< 0.001
Triglyceride (TG), mmol/L	1.18 ± 0.44	1.29 ± 0.46	1.09 ± 0.41	< 0.001
Low density lipoprotein (LDL-C), mmol/L	2.61 ± 0.63	2.67 ± 0.64	2.56 ± 0.61	< 0.001
High density lipoprotein (HDL-C), mmol/L	1.31 ± 0.21	1.23 ± 0.16	1.37 ± 0.22	< 0.001

*Note*: Age, BMI, TGs, LDL-C, and HDL-C are continuous variables and are presented as the mean ± standard error. Family history of diabetes and physical activity are categorical variables and are presented as numbers (percentages)

### Association between GRS and dyslipidaemia

The mean value of GRS in all participants was 1.33 (SD: 0.34). The overall association was significant between GRS and dyslipidaemia, with a crude *HR* (95% *CI*) of 1.366 (1.187, 1.572) and an adjusted *HR* (95% *CI*) of 1.353 (1.172, 1.561) (Table 2). Then, the GRS was divided into quartiles. Compared with Q1, subjects in the Q2, Q3, and Q4 groups had adjusted *HR*s (95% *CI*) of 1.043 (0.900, 1.210), 1.188 (1.028, 1.374), and 1.229 (1.069, 1.412), respectively, when adjusted for age, family history of diabetes, physical activity, BMI, and blood lipid indicators. The significant association suggested that the risk of developing dyslipidaemia steadily increased as the GRS increased. By the same token, adjusted and crude *HR*s showed the same constant increment in the training set and testing set.

**Table 2:** Association between GRS and incidence of dyslipidaemia

	Subjects	Crude *HR*s (95%*CI*)	Adjusted *HR*s (95%*CI*)
**Total population**			
Q1	900	1.00 (reference)	1.00 (reference)
Q2	898	1.110 (0.958, 1.287)	1.043 (0.900, 1.210)
Q3	900	1.244 (1.077, 1.437)	1.188 (1.028, 1.374)
Q4	898	1.276 (1.111, 1.466)	1.229 (1.069, 1.412)
Continuous GRS	3596	1.366 (1.187, 1.572)	1.353 (1.172, 1.561)
*P*_for trend_		< 0.001	0.001
**Training set**			
Q1	633	1.00 (reference)	1.00 (reference)
Q2	638	0.996 (0.834, 1.188)	1.023 (0.855, 1.223)
Q3	624	1.182 (0.995, 1.404)	1.166 (0.979, 1.388)
Q4	622	1.207 (1.023, 1.424)	1.213 (1.028, 1.433)
Continuous GRS	2517	1.337 (1.129, 1.584)	1.318 (1.110, 1.565)
*P*_for trend_		0.006	0.008
**Testing set**			
Q1	267	1.00 (reference)	1.00 (reference)
Q2	260	1.456 (1.112, 1.907)	1.081 (0.820, 1.425)
Q3	276	1.405 (1.080, 1.827)	1.225 (0.940, 1.596)
Q4	276	1.454 (1.129, 1.874)	1.273 (0.986, 1.643)
Continuous GRS	1079	1.432 (1.113, 1.843)	1.466 (1.127, 1.907)
*P*_for trend_		0.009	0.040

*Note*: GRS is divided into four groups. Q1, Q2, Q3, Q4 represent the first, second, third, fourth quartile of GRS, respectively. Adjusted *HR*s adjust for the following covariates: age, family history of diabetes, physical activity, BMI, TG, HDL-C, LDL-C

*Abbrevations*: *HR* hazard ratio, GRS genetic risk score

### Development and evaluation of the conventional models

**Fig. 1:**
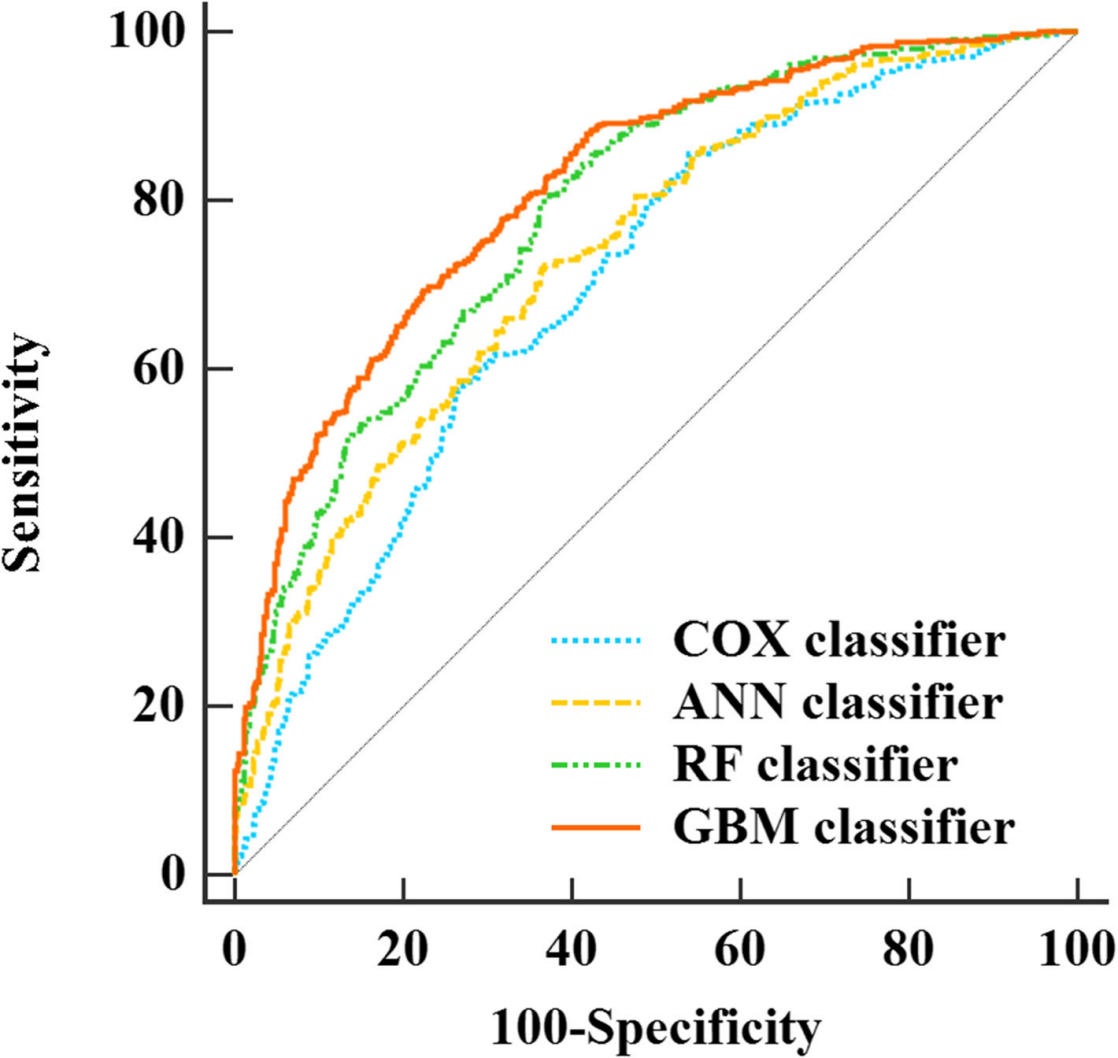
Receiver-operating characteristic curves of conventional models with four classifiers. Abbreviations: ANN: artificial neural network; RF: random forest; GBM: gradient boosting machine

In the training set, the 14 reported predictors were analysed using simple Cox regression, and 8 variables (age, family history of diabetes, physical activity, WC, BMI, TGs, HDL-C, and LDL-C) were statistically significantly related to dyslipidaemia. Eventually, the conventional models were composed of age, family history of diabetes, physical activity, BMI, TGs, HDL-C, and LDL-C (Table 3, above), considering the collinearity between WC and BMI. It is worth noting that there was no collinearity among TG, HDL-C, and LDL-C. The AUCs and their differences of the 4 conventional models with different classifiers are shown in Fig. 1 and Table 4. In the testing set, the AUCs of the conventional models with the Cox, ANN, RF, and GBM classifiers were 0.702(0.673, 0.729), 0.736(0.708, 0.762), 0.787 (0.762, 0.811), and 0.816(0.792, 0.839), respectively, indicating that the conventional models showed quite high performance in predicting dyslipidaemia, especially the model with the GBM classifier. In addition, concerning that it may be not practical to use blood lipid indicators to predict dyslipidaemia. The AUCs of the prediction model without the blood lipid index were calculated for the conventional and conventional+GRS model, and the AUCs were 0.553 (0.523, 0.583) and 0.569 (0.539, 0.598), respectively, when using the Cox classifier. The prediction model using machine learning methods showed the similar poor performance (see Table S4).

**Table 3:** Multiple Cox regression analysis on significant factors of developing dyslipidaemia in training set

Variables	*β*	S.E.	Wald	*P*	*HR* (95%*CI*)
**Conventional model**					
Age	0.005	0.003	3.017	0.082	1.005(0.999, 1.010)
Family history of diabetes	0.194	0.125	2.429	0.119	1.215(0.951, 1.551)
Physical activity					
Low	Reference				
Moderate	0.793	0.080	99.087	< 0.001	2.210(1.890, 2.583)
High	0.324	0.071	20.810	< 0.001	1.383(1.203, 1.590)
BMI	0.016	0.010	2.777	0.096	1.016(0.997, 1.036)
TG	0.292	0.074	15.609	< 0.001	1.339(1.158, 1.548)
HDL-C	−2.103	0.196	114.907	< 0.001	0.122(0.083, 0.179)
LDL-C	0.284	0.052	29.792	< 0.001	1.329(1.200, 1.472)
**Conventional + GRS model**					
Age	0.005	0.003	2.887	0.089	1.005(0.999, 1.010)
Family history of diabetes	0.198	0.125	2.517	0.113	1.219(0.954, 1.557)
Physical activity					
Low	Reference				
Moderate	0.802	0.080	101.097	< 0.001	2.230(1.907, 2.607)
High	0.328	0.071	21.347	< 0.001	1.389(1.208, 1.596)
BMI	0.017	0.010	2.998	0.083	1.017(0.998, 1.037)
TG	0.281	0.074	14.410	< 0.001	1.325(1.146, 1.532)
HDL-C	−2.095	0.195	114.889	< 0.001	0.123(0.084, 0.180
LDL-C	0.286	0.052	29.968	< 0.001	1.330(1.201, 1.474)
Weighted GRS	0.276	0.088	9.925	0.002	1.318(1.110, 1.565)

*Note*: The predictors of the conventional model are variables that are significantly associated with dyslipidaemia in simple Cox regression analysis. GRS is added to the conventional model to construct the conventional+GRS model

*Abbreviations*: *BMI* body mass index, *TG* triglyceride, *HDL-C* high density lipoprotein, *LDL-C* low density lipoprotein, *GRS* genetic risk score

**Table 4:** Performance of the conventional and conventional+GRS models in predicting dyslipidaemia

	AUC	△AUC	Continuous NRI, %	IDI, %
Cox				
Conventional model	0.702(0.673, 0.729)			
Conventional+GRS model	0.707(0.679, 0.734)	0.0049(*P* = 0.0549)	25.6 (13.8, 35.8)^*^	2.3 (1.1, 3.7)^*^
ANN				
Conventional model	0.736(0.708, 0.762)			
Conventional+GRS model	0.754(0.727, 0.779)	**0.0183(*P*** = 0.0031)^*^	7.8 (−2.7, 18.5)	1.0 (−0.3, 2.4)
RF				
Conventional model	0.787 (0.762, 0.811)			
Conventional+GRS model	0.810 (0.762, 0.811)	**0.0230(*P*** = 0.023)^*^	14.1 (1.1, 26.1)^*^	2.5 (0.5, 4.2)^*^
GBM				
Conventional model	0.816(0.792, 0.839)			
Conventional+GRS model	0.831(0.808, 0.853)	**0.0151(*P*** = 0.0135)^*^	18.1 (4.4, 27.2)^*^	1.8 (0.1, 3.5)^*^

*Abbreviations*: *AUC* area under receiver operating characteristic curve, *△AUC* difference between AUC of conventional model and conventional+GRS model, *NRI* net reclassification improvement, *IDI* integrated discrimination improvement, *ANN* artificial neural network, *RF* random forest, *GBM* gradient boosting machine

^*^Statistically significant values, *P* < 0.05

### Development and evaluation of conventional models with GRS

**Fig. 2:**
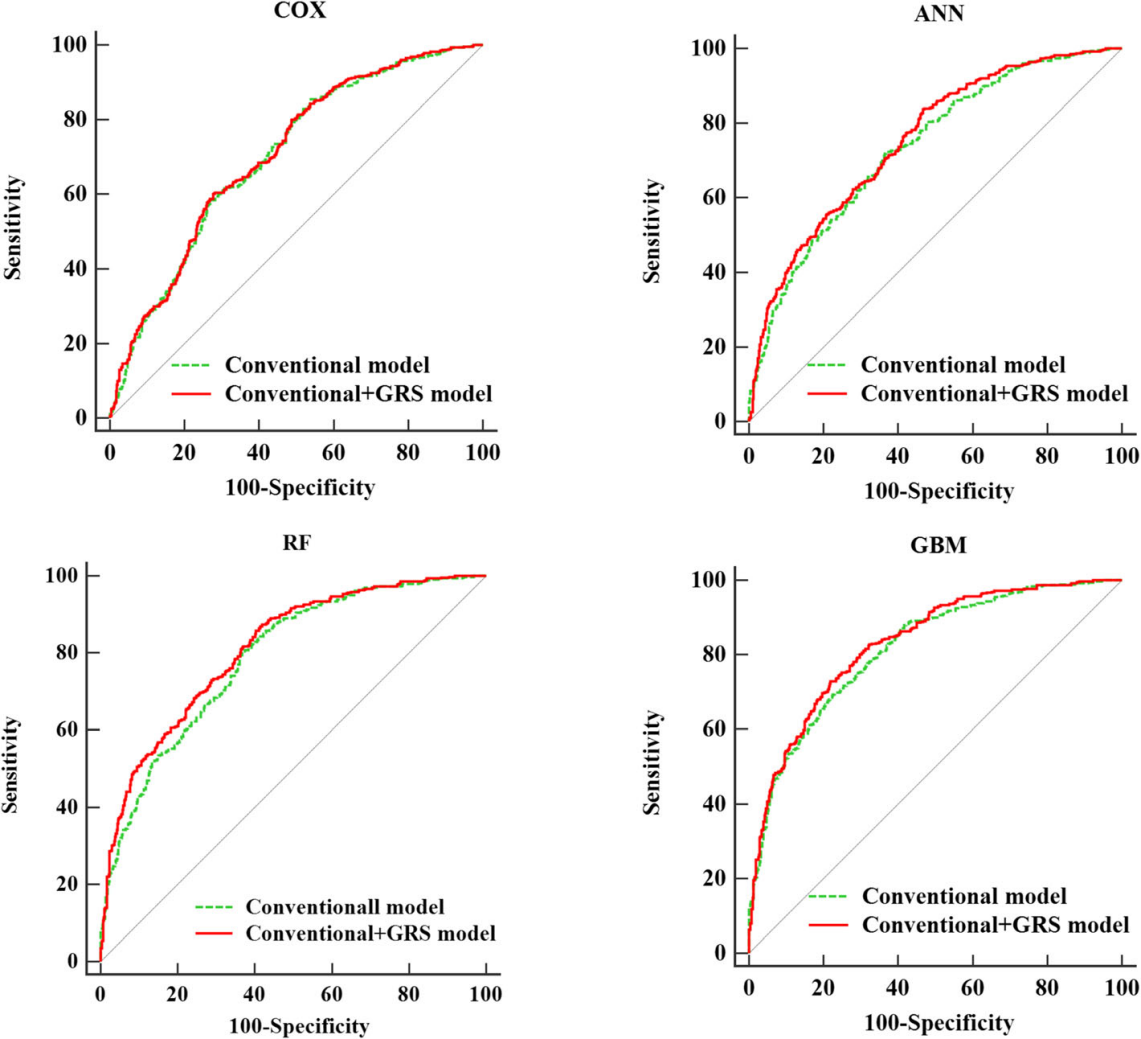
Receiver-operating characteristic curves of conventional model and conventional+GRS model with four classifiers. Note: Age, family history of diabetes, physical activity, BMI, TG, HDL-C, and LDL-C are included in the conventional model; the conventional+GRS model includes age, family history of diabetes, physical activity, BMI, TG, HDL-C, LDL-C and GRS. Abbreviations:ANN: artificial neural network; RF: random forest; GBM: gradient boosting machine; BMI: body mass index; TG: triglyceride; HDL-C: high density lipoprotein; LDL-C: low density lipoprotein; GRS: genetic risk score

The conventional+GRS model combined conventional factors and the GRS (Table 3, below). Table 4 shows the differences in discrimination between the conventional model and conventional+GRS model. In the case of using the Cox classifier, the addition of GRS improved the predictive ability of the conventional model in a limited way. The conventional model showed moderate discrimination, and the AUC increased slightly with the addition of GRS to 0.707 (0.679, 0.734); the difference in AUC was 0.0049 but was not statistically significant at *P* = 0.0549. Notwithstanding, the addition of GRS resulted in a statistically significant continuous NRI of 25.6% (13.8, 35.8%) and IDI of 2.3% (1.1, 3.7%). For the ANN classifier, the addition of GRS increased the AUC to 0.754 (0.727, 0.779); the difference in the AUC was 0.0183 (*P* = 0.0031). Nevertheless, the continuous NRI and IDI were 7.8% (− 2.7, 18.5%) and 1.0% (− 0.3, 2.4%), respectively, presenting no statistical significance. Additionally, the conventional+GRS model with the RF and GBM classifier resulted in significant improvements (NRI for RF: 14.1% (1.1, 26.1%); IDI for RF: 2.5% (0.5, 4.2%); NRI for GBM: 18.1% (4.4, 27.2%); IDI for GBM: 1.8% (0.1, 3.5%)), demonstrating the competent progress of GRS in predicting dyslipidaemia. The discrimination of the prediction model with RF classifier showed significant improvements better than the GBM classifier when adding GRS into the conventional model. Figure 2 provides the receiver operating characteristic curves (ROCs) for the conventional and conventional+GRS models with different classifiers. The results suggested that the addition of GRS could improve the prediction performance of the conventional models in some aspects in most classifiers. In addition, the GBM classifier presented the best performance with an AUC of 0.831 (0.808, 0.853) of all the models.

**Fig. 3:**
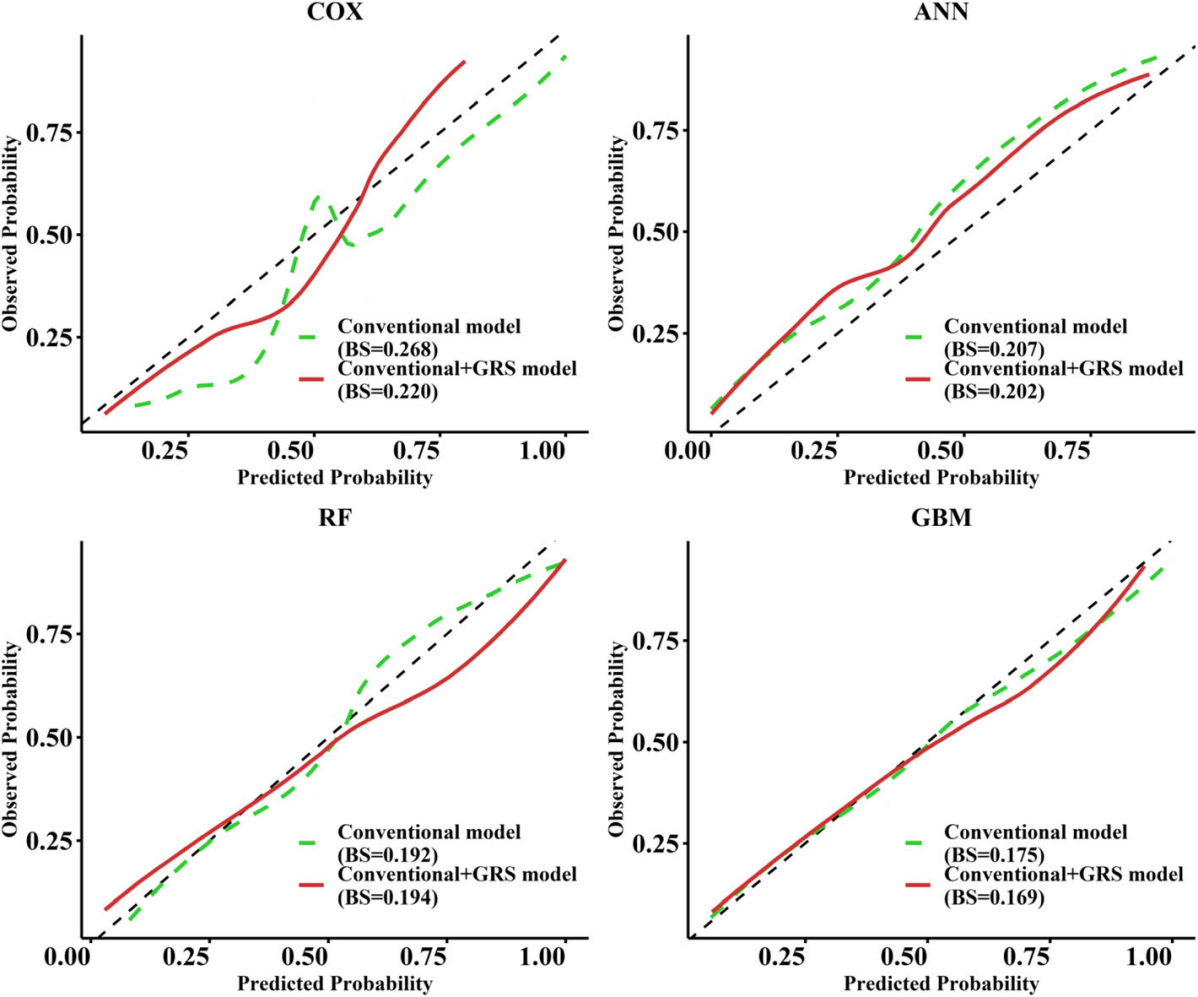
Calibration curves of conventional model and conventional+GRS model with four classifiers. Note: The dotted grey line is an ideal curve, indicating that the predicted probability is consistent with the observed one. The lower the Brier score, the closer the calibration curve is to the ideal curve. Abbreviations:ANN: artificial neural network; RF: random forest; GBM: gradient boosting machine; BS: Brier score

Figure 3 demonstrates the calibrations of the conventional and conventional+GRS models. The calibration curves of the conventional+GRS models were closer to the reference line (dotted grey line) than those of the conventional models. The Brier scores, which can be considered a “calibration” measure of a set of probabilistic predictions, also declined with the addition of GRS (Cox declined 0.048, ANN classifier slightly declined 0.005, and GBM declined 0.006), indicating conventional models were provided with better calibration when incorporating GRS (The lower the Brier score value, the better the prediction calibration). Other statistics, such as sensitivity and specificity, were provided in Table S3. These metrics provided further evidence that the predictive ability of the models was improved by adding GRS.

## Discussion

To our knowledge, this is the first study to explore the utility of genetic factors in the prediction of dyslipidaemia in resource-limited areas based on a prospective study. The results of this study suggested that individuals in higher GRS quartiles displayed an increased risk of dyslipidaemia onset compared to those with the lowest quartile of GRS. The conventional models were constructed with Cox, ANN, RF, and GBM classifiers. The model with the GBM classifier significantly outperformed the other classifiers. More importantly, the accession of GRS convincingly improved the capability of conventional models in predicting dyslipidaemia, implying that genetic factors play a meaningful role in predicting the occurrence of dyslipidaemia.

This study elaborated the correlation between genetic factors (GRS) and dyslipidaemia by dividing the GRS into quartiles. A previous study divided all participants into 3 groups according to GRSs of LDL-C, HDL-C, and TG, and showed that the group with the highest GRS in HDL-C, LDL-C, and TG all presented higher lipid levels than the group with the lowest GRS [27]. Similarly, in this study, the results suggested that the higher the GRS was, the higher the risk of developing dyslipidaemia, regardless of age, family history of diabetes, physical activity, BMI, and blood lipid indicators. Although not every *HR* was statistically significant, dyslipidaemia risk increased within each quartile of GRS, and a similar trend was observed in the training set and testing set. The above demonstrated statistical results of the significantly enhanced incidence of dyslipidaemia risk in rural populations with incremental GRS.

The results showed that the conventional model consisting of seven variables presented the best predictive performance when the GBM classifier was used. Previous studies revealed a dozen variables as predictors of dyslipidaemia [16, 18]. However, 8 variables showed statistical significance in the simple Cox regression analysis, and 7 of them were finally included in the conventional model. Based on the results, simple Cox regression tagged baseline lipoproteins including TG, HDL-C, and LDL-C as predictors, which was a reasonable result because plasma lipoproteins currently lead to abnormal future blood lipids. In addition, the *HR*s of these predictors were comparable to those of other association studies [9, 30–34]. Correspondingly, the *HR*s of these 7 variables were also consistent with those in early published studies of dyslipidaemia prediction models [16, 18, 19]. Notably, the three serum lipid parameters showed no collinearity. The findings indicated that the GBM classifier could predict the incidence of dyslipidaemia better, which was confirmed in our previous study [35]. This might be because the GBM classifier could address the intricate relationship between predictors and dyslipidaemia.

Considering the moderate but strong association between GRS and dyslipidaemia, the increased benefit of GRS was then determined in predicting the occurrence of dyslipidaemia. The performance of the conventional model improved significantly when using RF and GBM classifiers, both in terms of discrimination and net improvement metrics. In contrast, the model using the ANN classifier showed less obvious improvement with the inclusion of GRS, with slightly incremental but insignificant NRI and IDI (*P* > 0.05). Nevertheless, improvements in AUC were observed in the Cox, RF, and GBM classifiers, both numerically and statistically. As was shown in an earlier study [26], in the transition from childhood to adulthood, the predictive power of GRSs on HDL-C, LDL-C, and TG is valuable in predicting adulthood lipid levels. Individuals with any abnormal lipid index can be defined as having dyslipidaemia; thus, GRS might have a predictive effect on dyslipidaemia, and the results partially confirm this. Furthermore, the results also suggested that the application of the machine learning technique might perform better in disease prediction than the statistical method, which was consistent with the results of previous studies [36, 37]. Similarly, the elevation of other statistical (Table S3) values showed that GRS played a relatively important role in dyslipidaemia prediction. Principally, the results of this study revealed that GRS could be a possible predictor of the occurrence of dyslipidaemia.

As was demonstrated in a previous study [38], the disclosure of coronary heart disease risk estimates indicated that the inclusion of genetic risk information could reduce the levels of LDL-C compared to the disclosure based on conventional risk factors only. Genetic risk information for common diseases could be incorporated into the conventional predictive model and used to guide treatment. Considering how lipid levels influence CVD [39, 40], it is reasonable to infer that the addition of the GRS into the prediction model of dyslipidaemia might help individuals prevent abnormal blood lipid levels and thus contribute to the prevention of cardiovascular events.

## Study strength and limitations

This research clarified the crucial impact of genetic information in predicting dyslipidaemia in rural areas, signifying a certain guiding role of gene information in the prevention and treatment of clinical dyslipidaemia. To some extent, the research indicated that the machine learning method might have certain advantages in the construction of the disease prediction model. Additionally, a cohort study was used to construct and validate the conventional model and to analyse the relationship between genetic factors and dyslipidaemia, making the results more convincing.

However, several limitations need to be addressed. First, the integration of the four lipid measurements (TC, TG, LDL-C, and HDL-C) into dyslipidaemia might gloss over the ability of genetic information in each lipid index. However, better performances of the GRS-integrated-model demonstrated that genetic information was impressive in blood lipids, providing a foundation for follow-up studies about genetic factors and lipid levels. Another limitation is that the Brier score failed to test statistically in assessing the calibration of models, although the value had declined. Third, there were also limitations in screening out predictors with the Cox regression model. The Cox model was restricted by its very strict application conditions, such as proportional hazard assumptions. Only those variables that meet the strict conditions would be considered for screening with the Cox model, and thus some possible predictors might be ignored before being filtered. Last, the representation might be limited because the recruited subjects only came from rural areas in China, so the extrapolation of the conclusions was restricted by the lack of external validation. However, 30% of subjects were randomly selected to conduct internal verification to increase the credibility of the study.

## Conclusion

Based on the prospective cohort study, eight dyslipidaemia prediction models with and without the genetic factor (GRS) were developed and evaluated. The conventional models included age, family history of diabetes, physical activity, BMI, TGs, HDL-C, and LDL-C, which showed better performance in predicting dyslipidaemia, especially with the GBM classifier. After adding genetic factors, the prediction performance of the conventional models was effectively enhanced. This study provided an alternative plan for the screening of dyslipidaemia, which might help in the diagnosis and prevention of clinical dyslipidaemia, allow us to screen for genetic risk early in life and help individuals prevent dyslipidaemia in advance.

## Supplementary Information

**Additional file: 1 Table S1.** The weight of 21 SNPs based on our population. **Table S2.** Simple Cox regression analysis of risk factors for developing dyslipidaemia in the raining set. **Table S3.** Other statistics of the conventional model and conventional+GRS model. **Table S4.** Performance of models without blood lipid indexes. **Fig. S1.** Flowchart of the study population and model constitution and evaluation.

## Data Availability

The data are available from the corresponding author with reasonable justification.

## Abbreviations

GRS: genetic risk score
AUC: Area under ROC curve
NRI: Net reclassification index
IDI: Integrated discrimination index
CVD: Cardiovascular diseases
TG: Triglyceride
HDL-C: High-density lipoprotein cholesterol
TC: Total cholesterol
LDL-C: Low-density lipoprotein cholesterol
SNPs: Single nucleotide polymorphisms
BMI: Body mass index
WC: Waist circumference
*HR*s: Hazard ratios
ANN: Artificial neural network
RF: Random forest
GMB: Gradient boosting machine
SD: Standard deviation
