# Supplementary material for: Genetic factors increase the identification efficiency of predictive models for dyslipidaemia: a prospective cohort study
Source: Lipids Health Dis. 2021 Feb 12;20:11. doi: 10.1186/s12944-021-01439-3 (PMC7881493; doi:10.1186/s12944-021-01439-3)
Supplement: Supplementary file 1 — Additional file: 1 Table S1. The weight of 21 SNPs based on our population. Table S2. Simple Cox regression analysis of risk factors for developing dyslipidaemia in the raining set. Table S3. Other statistics of the conventional model and conventional+GRS model. Table S4. Performance of models without blood lipid indexes. Fig. S1. Flowchart of the study population and model constitution and evaluation. [file 12944_2021_1439_MOESM1_ESM.docx]

**Table S1.** The weight of 21 SNPs based on our own population

| SNP | Risk / No-Risk Allele | *β* (weight) | *S.E.* | *P* value | *HR* (95%*CI*) |
| --- | --- | --- | --- | --- | --- |
| rs10889353 | **A**/C | 0.054 | 0.045 | 0.233 | 1.055(0.966,1.153) |
| rs11207995 | **A**/C | 0.048 | 0.045 | 0.289 | 1.049(0.960,1.146) |
| rs7518497 | **A**/C | 0.044 | 0.046 | 0.334 | 1.045(0.956,1.143) |
| rs780092 | **G**/A | 0.095 | 0.037 | 0.011 | 1.100(1.022,1.183) |
| rs10045497 | **A**/C | 0.088 | 0.036 | 0.015 | 1.091(1.017,1.171) |
| rs11216126 | **A**/C | 0.061 | 0.041 | 0.142 | 1.063(0.980,1.152) |
| rs1160985 | **C**/T | 0.057 | 0.040 | 0.156 | 1.059(0.978,1.146) |
| rs17119975 | **T**/C | 0.075 | 0.041 | 0.066 | 1.078(0.995,1.169) |
| rs183786 | **C**/T | 0.016 | 0.037 | 0.668 | 1.016(0.945,1.093) |
| rs328 | **G**/C | 0.055 | 0.063 | 0.385 | 1.056(0.934,1.194) |
| rs3764261 | **C**/A | 0.113 | 0.050 | 0.025 | 1.120(1.015,1.236) |
| rs3943077 | **G**/A | 0.023 | 0.037 | 0.526 | 1.024(0.953,1.100) |
| rs4417316 | **C**/T | 0.086 | 0.040 | 0.034 | 1.089(1.006,1.006) |
| rs507666 | **A**/G | 0.023 | 0.045 | 0.605 | 1.024(0.937,1.118) |
| rs603446 | **C**/T | 0.061 | 0.041 | 0.140 | 1.063(0.980,1.153) |
| rs651007 | **T**/C | 0.018 | 0.045 | 0.687 | 1.018(0.932,1.112) |
| rs651821 | **C**/T | 0.138 | 0.040 | 0.001 | 1.148(1.061,1.242) |
| rs6589566 | **G**/A | 0.105 | 0.044 | 0.016 | 1.110(1.020,1.209) |
| rs662799 | **G**/A | 0.131 | 0.040 | 0.001 | 1.140(1.054,1.234) |
| rs7396835 | **T**/C | 0.114 | 0.038 | 0.003 | 1.121(1.040,1.208) |
| rs964184 | **G**/C | 0.106 | 0.043 | 0.015 | 1.112(1.021,1.211) |

**Note:** COX regression is used to analyze the association of single SNP with incident dyslipidemia. The bold font represents the risk allele. SNP: single nucleotide polymorphism; *HR*: hazard ratio.

**Table S2.** Simple Cox regression analysis of risk factors for developing dyslipidaemia in the raining set

| Variables | *β* | **S.E.** | *Wald* | *P* value | *HR* (95%*CI*) |
| --- | --- | --- | --- | --- | --- |
| Age | 0.010 | 0.002 | 17.544 | **<0.001** | 1.010(1.005, 1.015) |
| Male | 0.001 | 0.065 | 0.001 | 0.982 | 1.001(0.882, 1.137) |
| Higher-educational level | -0.028 | 0.034 | 0.690 | 0.406 | 0.972(0.909, 1.039) |
| Smoking | -0.107 | 0.072 | 2.181 | 0.140 | 0.889(0.780, 1.036) |
| High-fat diet | -0.282 | 0.207 | 1.860 | 0.173 | 0.754(0.503, 1.131) |
| More vegetable and fruit intake | 0.066 | 0.061 | 1.189 | 0.275 | 1.069(0.948, 1.204) |
| Family history of hyperlipidemia | -0.194 | 0.109 | 3.126 | 0.077 | 0.824(0.665, 1.021) |
| **Family history of diabetes** | 0.252 | 0.123 | 4.216 | **0.040** | 1.287(1.012, 1.637) |
| More physical activity | 0.205 | 0.033 | 38.202 | **<0.001** | 1.227(1.150, 1.310) |
| WC | 0.018 | 0.003 | 31.416 | **<0.001** | 1.018(1.011, 1.024) |
| **BMI** | 0.058 | 0.009 | 42.778 | **<0.001** | 1.060(1.041, 1.078) |
| **TG** | 0.656 | 0.054 | 148.599 | **<0.001** | 1.972(1.734, 2.141) |
| **HDL-C** | -2.164 | 0.159 | 184.980 | **<0.001** | 0.115(0.084, 0.157) |
| **LDL-C** | 0.347 | 0.040 | 76.726 | **<0.001** | 1.415(1.309, 1.529) |

**Note:** Variables in normal font are those in a cross-sectional study published in 2012 [1], while variables in bold font are those in the Taiwan cohort study [2]. Sex was included in both two studies. All the significant *P* values were in bold.

**Table S3.** Other statistics of the conventional model and conventional+GRS model

| Model | Sensitivity, % | Specificity, % | Positive Predictive Value, % | Negative Predictive Value, % |
| --- | --- | --- | --- | --- |
| Conventional model | | | | |
| COX | 85.57 (82.08,88.49) | 46.00 (41.92,50.13) | 57.05 (53.38,60.64) | 79.18 (74.40,83.29) |
| ANN | 71.95 (67.72,75.84) | 63.54 (59.49,67.42) | 62.32 (58.18,66.30) | 72.99 (23.25,31.12) |
| RF | 80.49 (76.65,83.84) | 62.86 (58.80,66.76) | 64.50 (60.55,68.26) | 79.35 (75.33,82.89) |
| GBM | 69.92 (65.62,73.90) | 77.00 (73.34,80.30) | 71.81 (67.52,75.76) | 75.33 (71.64,78.69) |
| Conventional+GRS model | | | | |
| COX | 59.96 (55.47,64.29) | 72.23 (68.39,75.78) | 64.41 (59.81,68.76) | 68.28 (64.43,71.89) |
| ANN | 83.74 (80.11,86.83) | 53.32 (49.19,57.41) | 60.06 (56.27,63.73) | 79.64 (75.25,83.45) |
| RF | 86.99 (83.62,89.77) | 58.60 (54.49,62.60) | 63.79 (60.00,67.41) | 84.31 (80.33,87.63) |
| GBM | 72.97 (68.77,76.80) | 78.02 (74.41,81.27) | 73.57 (69.37,77.38) | 77.50 (73.87,80.76) |

ANN: artificial neural network; RF: random forest; GBM: gradient boosting machine.

**Table S4.** Performance of models without blood lipid indexes

|  | AUC | △AUC | Continuous NRI, % | IDI, % |
| --- | --- | --- | --- | --- |
| Cox |  |  |  |  |
| Conventional model | 0.553(0.523, 0.583) |  |  |  |
| Conventional+GRS model | 0.569(0.539, 0.598) | **0.0155(*P*=0.0055)** | 5.8 (-2.4, 19.1) | 0.8 (-0.2, 1.8) |
| ANN |  |  |  |  |
| Conventional model | 0.601 (0.571, 0.630) |  |  |  |
| Conventional+GRS model | 0.627 (0.597, 0.656) | **0.0259(*P*=0.0287)** | **15.9 (2.4, 22.0)** | **1.5 (0.4, 2.8)** |
| RF |  |  |  |  |
| Conventional model | 0.639 (0.610, 0.668) |  |  |  |
| Conventional+GRS model | 0.684 (0.656, 0.712) | **0.0449(*P*=0.0002)** | **9.8 (2.1, 22.6)** | **2.8 (1.2, 4.7)** |
| GBM |  |  |  |  |
| Conventional model | 0.653 (0.624, 0.682) |  |  |  |
| Conventional+GRS model | 0.691 (0.663, 0.719) | **0.0379(*P*=0.0007)** | **9.6 (1.6, 18.7)** | **2.5 (1.1, 4.1)** |

**Note:** The conventional model included age, family history of diabetes, and physicial activity. **Abbreviations:** AUC: area under receiver operating characteristic curve; △AUC: difference between AUC of conventional model and conventional+GRS model; NRI: net reclassification improvement; IDI: integrated discrimination improvement; ANN: artificial neural network; RF: random forest; GBM: gradient boosting machine.

^*^ Statistically significant values, *P* <0.05


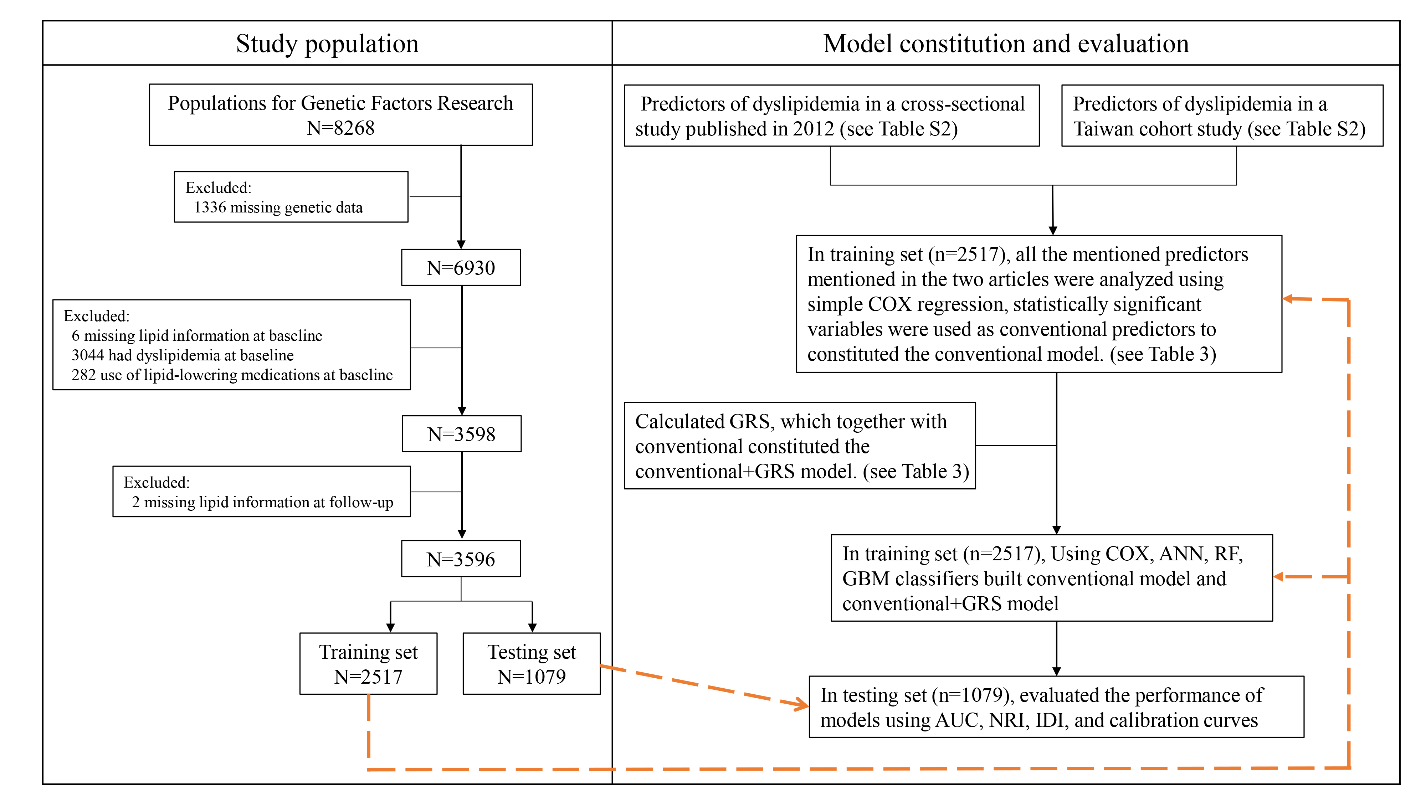


**Figure S1.** Flowchart of the study population and model constitution and evaluation. The orange dashed line indicates the data set used to perform the corresponding analysis

**References**

1. Wang CJ, Li YQ, Wang L, Li LL, Guo YR, Zhang LY, Zhang MX, Bie RH: **Development and evaluation of a simple and effective prediction approach for identifying those at high risk of dyslipidemia in rural adult residents.** *PLoS One* 2012, **7:**e43834.

2. Yang X, Xu C, Wang Y, Cao C, Tao Q, Zhan S, Sun F: **Risk prediction model of dyslipidaemia over a 5-year period based on the Taiwan MJ health check-up longitudinal database.** *Lipids Health Dis* 2018, **17:**259.
